# The Beta-Cell Function and Glucose Profile of Newly Diagnosed Acromegalic Patients with Normal Glucose Tolerance

**DOI:** 10.1155/2021/3666692

**Published:** 2021-12-07

**Authors:** Quanya Sun, Xiaoqing Li, Peili Chen, Lili Chen, Xiaolong Zhao

**Affiliations:** ^1^Department of Endocrinology, Huashan Hospital Fudan University, Shanghai, China; ^2^Department of Endocrinology and Metabolism, Shanghai Public Health Clinical Center, Fudan University, Shanghai, China

## Abstract

**Objectives:**

Untreated acromegaly is a nature model for unveiling the diabetogenic effects of GH. CGMS can uncover more glucose profile of acromegaly. This study aimed to evaluate the insulin resistance (IR), *β*-cell function, and glycemic spectrum of patients with newly diagnosed acromegaly with normal glucose tolerance (NGT).

**Methods:**

This study was conducted in Huashan Hospital from January 2015 to February 2019. Eight newly diagnosed acromegalic patients without history of diabetes and eight age- and gender-matched healthy subjects were enrolled. All participants underwent oral glucose tolerance test (OGTT) and 72 h continuous glucose monitoring (CGM). Parameters on *β*-cell function and IR were calculated. Mean blood glucose (MBG) in 24 hours was adopted for the evaluation of the glycemic level, and standard deviation of blood glucose (SDBG) and mean amplitude of glycemic excursion (MAGE) were used for glucose fluctuation.

**Results:**

HbA1c in the acromegaly group was significantly higher than in the control. During OGTT, glucose peaked at 60 min in acromegaly and at 30 min in controls. After glucose load, the acromegaly group had significantly higher insulin levels than controls, especially in 120 min and 180 min. Both insulin sensitivity index and disposal index after glucose load of acromegaly were significantly lower than those of controls. Moreover, acromegalic subjects had significantly higher MBG than controls.

**Conclusions:**

The newly diagnosed acromegalic patients with NGT were characterized by IR and impaired *β*-cell function after glucose load. CGM showed that MBG of NGT acromegaly patients was higher than that of normal people.

## 1. Introduction

Acromegaly is caused by excessive secretion of the growth hormone (GH), resulting in increased production of insulin-like growth factor-1 (IGF-1), which is responsible for clinical manifestations and for the systemic complications associated with increased mortality [1].

The incidence of glucose intolerance in acromegaly is very common. Studies have shown that 12% to 52.5% of acromegalic patients have diabetes [2–4], while 15% to 36% of acromegalic patients have impaired glucose tolerance [2, 4–6]. A meta-analysis has showed that the all-cause mortality risk in patients with acromegaly was 1.72 (95% confidence interval: 0.62–1.83) [7]. Studies have shown that hyperglycemia is probably a risk factor for the poor prognosis of acromegaly independently of cardiovascular disease [8–10].

Untreated acromegaly is a nature model for unveiling the diabetogenic effects of GH. Pathologic increases in plasma growth hormone concentrations in acromegaly cause hyperinsulinemia and insulin resistance [11–13]. Although some studies have described the glucose profile, beta-cell function, and insulin sensitivity in patients with acromegaly, most of them enrolled the GH patients under antidiabetic medicine which may interfere beta-cell function and insulin sensitivity analysis [14, 15]. In our study, we enrolled the newly diagnosed acromegalic patients without any intervention to the beta-cell function and insulin sensitivity evaluation.

On the contrary, previous studies on glucose metabolism in acromegaly mainly use traditional monitoring parameters, such as fasting glucose and oral glucose tolerance test. So far, there has been no study to conduct comprehensive blood glucose monitoring in patients with acromegaly. The continuous glucose monitoring system (CGMS) can provide much more glycemic information, including magnitude, duration, and frequency of blood glucose levels, which helps better understand the properties of dynamic blood glucose levels throughout the day [16]. In the current study, we used CGM to reveal the blood glucose profile of newly diagnosed acromegalic patients compared with normal controls.

## 2. Materials and Methods

### 2.1. Patients

**Figure 1:**
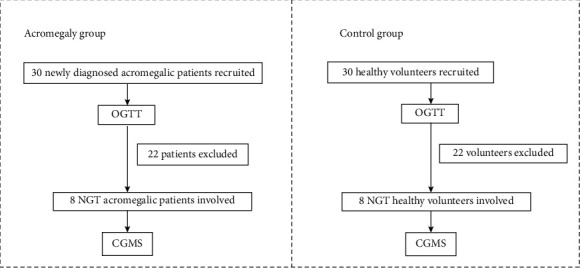
Research flowchart.

The current study was conducted in Huashan Hospital, Shanghai, from January 2015 to February 2019. The study was approved by the Institutional Review Broad of Huashan Hospital, Fudan University, and was registered in the clinical trial (NCT02574793). In all, 8 newly diagnosed acromegalic patients (2 males and 6 females) and 8 age- and gender-matched healthy controls were enrolled.  Figure 1 shows the recruitment and selection and exclusion process. Written consent was obtained from all participants.

The inclusion criteria of patients with acromegaly were as follows: (1) newly diagnosed acromegaly: the diagnosis criteria of acromegaly were based on the clinical features of acromegaly, failure of GH suppression to below 1 *μ*g/L in response to a 75 g oral glucose load, serum IGF-1 level above the age-appropriate reference range, and radiologic evidence of a pituitary tumor; (2) without inappropriate secretion of other pituitary hormones which could affect glucose metabolism, including TSH and ACTH; (3) OGTT validated normal glucose tolerance according to the World Health Organization (WHO) criteria; (4) no drugs affecting glucose metabolism were used in previous 3 months. The acromegaly group exclusion criteria were as follows: (1) multiple hormones secreting pituitary adenomas; (2) previous history of pituitary surgery, radiotherapy, or a history of somatostatin analogue treatment for acromegaly; (3) the age was younger than 18 years or older than 60 years; (4) patients with incomplete medical history data. The inclusion criteria of healthy controls were as follows: (1) GH can be suppressed to below 1 *μ*g/L in response to a 75 g oral glucose load, and plasma IGF-1 levels were in the age-appropriate reference range; (2) OGTT validated normal glucose tolerance according to the WHO criteria; (3) no drugs affecting glucose metabolism were used for previous 3 months; (4) with normal kidney and liver function and no abnormal signs or symptoms suggesting other diseases. The exclusion criteria for the healthy control group were as follows: (1) was previously diagnosed with diabetes or prediabetes; (2) currently taking drugs that affect blood glucose, including glucocorticoids and glucagon-like peptide-1 receptor agonists; (3) the age was younger than 18 years or older than 60 years.

After fasting for at least 8 hours, all participants underwent OGTT (75 g glucose). Blood samples were collected at 0, 30, 60, 90, and 120 min for the measurement of glucose, insulin, and GH. Normal glucose tolerance (NGT) was defined as fasting blood glucose (FBG) below 6.1 mmol/L and 2-hour plasma blood glucose below 7.8 mmol/L according to the WHO criteria [17].

The blood glucose (BG) levels of subjects were monitored continuously for 72 hours by CGMS (Medtronic MiniMed, MMT-7102). The criteria of CGM accuracy were as follows: at least 4 fingerstick BG values were input for sensor calibration each day with mean absolute differences # 28% and a correlation coefficient $ 0.79. Subjects had complex meals containing 50% carbohydrate, 15% protein, and 35% fat provided by the nutritional department before and during the study. The daily calorie intake was 25 kcal/kg. Meals were served at 6:30 to 7:30 AM, 11:00 AM to 12:00 PM, and 5:00 to 6:00 PM. The participants were required to finish each meal within 30 minutes.

The glucose profiles obtained from CGMS were downloaded using MiniMed Solution Software (Medtronic MiniMed). Glycemia was calculated as the 24-hour mean BG value (MBG). Intraday glycemic excursions were calculated as standard deviation of BG (SDBG) and the mean amplitude of glycemic excursion (MAGE) using CGMS analysis software [18].

### 2.2. Assays

BG was measured by Hitachi 7600 Biochemical Analyzer (Tokyo, Japan). Insulin was measured by the chemiluminescence immunoassay (ADVIA Centaur XP, Siemens, USA). HbA1c was detected with high-performance liquid chromatography (Tosoh HLC-723 G8 HPLC Analyzer, Japan).

GH was measured by a two-site chemiluminescent immunometric assay AutoDELFIA^®^ hGH (PerkinElmer Life and Analytical Sciences, Wallac Oy), intra-assay CV: 5.3–6.5%, interassay CV: 5.7–6.2%, and sensitivity: up to 0.01 *μ*g/l (0.026 mU/l).

IGF-1 was measured with IMMULITE 2000 solid-phase, enzyme-labeled chemiluminescent immunometric assay (Siemens Healthcare Diagnostic Products Limited, UK), with an age-specific normal range (1–6 years: 49–327 *μ*g/l; 7–11 years: 57–551 *μ*g/l; 12-13 years: 143–850 *μ*g/l; 14–16 years: 220–996 *μ*g/l; 17-18 years: 163–731 *μ*g/l; 19-20 years: 127–483 *μ*g/l; 21–35 years: 115–358 *μ*g/l; 36–50 years: 94–284 *μ*g/l; >50 years: 55–238 *μ*g/l; intra-assay CV: 2.3–3.5%; interassay CV: 7.0–7.1%; sensitivity: 20 *μ*g/l).

### 2.3. Insulin Sensitivity and Beta-Cell Function Evaluation

HOMA-beta was calculated as (20 × INS_0_)/(BG_0_ − 3.5) [19]. HOMA-IR was calculated as INS_0_ × BG0/22.5 [19]. BG_0_/INS_0_ was calculated as fasting glucose divided by fasting insulin. AUC_BG_ (the area under the glucose curve) was calculated as (BG_0_ + BG_30_) × 15 + (BG_30_ + BG_60_) × 15 + (BG_60_ + BG_120_) × 30 + (BG_120_ + BG_180_) × 30. AUC_INS_ (the area under the insulin curve) was calculated as (INS_0_ + INS_30_) × 15 + (INS_30_ + INS_60_) × 15 + (INS_60_ + INS_120_) × 30 + (INS_120_ + INS_180_) × 30. Insulin secretion index was calculated as AUC_INS_/AUC_BG_ [20]. IS_OGTT_ (insulin sensitivity after glucose load) was calculated as 10,000/SQRT (BG_0_ × INS_0_ × BG_mean_ × INS_mean_) [21]. For beta-cell function after glucose load, ISSI2 (insulin secretion-sensitivity index-2) was calculated as (AUC_Ins_/AUC_BG_) × ISO_GTT_ [20].

### 2.4. Statistical Analysis

Normally distributed data were expressed as means ± SD, whereas variables with a skewed distribution were reported as median (interquartile range). Two-tailed *t*-test was used to compare normal distribution data, and Mann–Whitney *U* rank test was used for skewed distribution data. Statistical analysis was performed with SPSS 23.0 software.

## 3. Results

### 3.1. Clinical Features of Study Subjects

As shown in Table 1, there were no significant differences between the acromegaly and control group in age and BMI. HbA1c in the acromegaly group was significantly higher than in the control (5.73 ± 0.30 vs. 5.35 ± 0.23, *P* = 0.025). The nadir GH (*μ*g/L) during OGTT of the acromegaly group was significantly higher than the control (6.39 (2.73–12.12) vs. 0.34 (0.12–0.48), *P* = 0.001).

### 3.2. OGTT and Insulin Releasing Test

**Figure 2:**
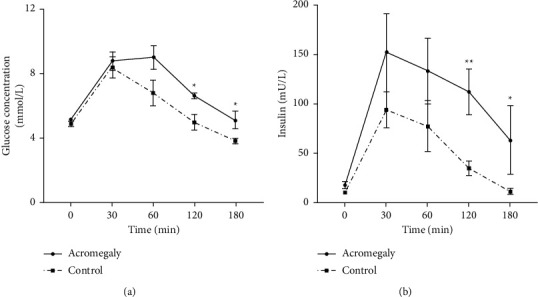
Glucose and insulin levels during OGTT. ^*∗*^*P* < 0.05, compared with the control group and ^*∗∗*^*P* < 0.01, compared with the control group.

As shown in Figure 2(a), peak blood glucose occurred at 30 min in the control group while at 60 min in the acromegaly group. Furthermore, the glucose levels at 120 min and 180 min in the acromegaly group were significantly higher than those in the control (*P* < 0.05). In the acromegaly group, the time of the glucose peak (60 min) was inconsistent with that of the insulin peak (30 min). Compared with the control group, the acromegaly group had higher insulin levels after glucose loading, especially in 120 min and 180 min (*P* < 0.05, Figure 2(b)).

### 3.3. Beta-Cell Function and Insulin Sensitivity

In the fasting stage, there was no significant difference in HOMA-beta and HOMA-IR and the insulin sensitivity index (BG_0_/INS_0_) between acromegaly and control groups (*P* > 0.05, Table 2). The disposition index (DI, calculated as (BG_0_/INS_0_) × HOMA-beta)) describing the beta-cell sensitivity-secretion relationship was not significantly different in acromegaly and control groups (61.18 (58.89–68.44) vs. 74.06 (61.17–82.65), *P* > 0.05, Table 2).

After glucose load, the insulin sensitivity index (IS_OGTT_) of the acromegaly group was significantly lower than that of the control group (50.25 (34.76–69.50) vs. 104.63 (67.14–129.58), *P* = 0.012, Table 2). The disposition index (DI, also called ISSI2, calculated as (AUC_Ins_/AUC_BG_) × ISO_GTT_) in acromegaly was significantly lower than in the control group (551.32 (520.87–684.55) vs. 716.12 (588.83–779.83), *P* = 0.046, Table 2).

### 3.4. Glycemic Excursions in Patients with Acromegaly

**Figure 3:**
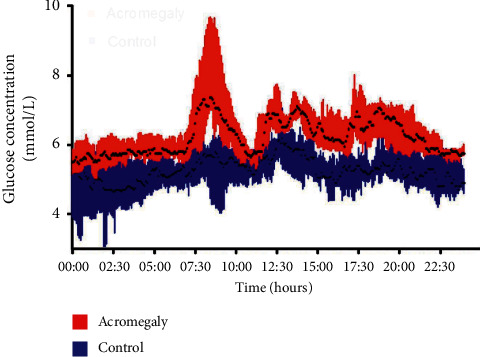
The 24-hour glucose profile of two groups. Data are medians (25^th^–75^th^ percentile).

Table 3 and Figure 3 show the results of CGM measurements. Compared with the control group, acromegaly had significantly higher MBG ((6.46 ± 0.44 vs. 5.31 ± 0.49) mmol/L, *P* < 0.001) and percentage of time for blood glucose level ≥ 7.8 mmol/L (PT7.8 (8.13 ± 6.49)% vs. (1.75 ± 2.66)%, *P* = 0.022).The glucose curve of CGMS in acromegaly is higher than that of the control group. There were no significant differences in glycemic variability parameters between acromegaly and control groups (*P* > 0.05, Table 3).

## 4. Discussion

Our study demonstrated hyperinsulinemia and reduced insulin sensitivity (IS_OGTT_) of acromegalic patients with NGT compared with healthy subjects. IS_OGTT_ obtained from oral glucose tolerance testing was indicated highly correlated with the rate of whole-body glucose disposal during the euglycemic insulin clamp [21]. Previous studies also showed hyperinsulinemia and insulin resistance in acromegaly [11–13]. Cerasi and Luft revealed that active acromegalic patients had hyperinsulinemia, but the response to a standardized glucose infusion varied greatly, which may represent different and consecutive stages in the development of diabetes in acromegaly [13]. Similar studies from Mayo Clinic also found that insulin secretion was increased basally and after glucose loading in acromegaly [11, 12]. Otherwise, the euglycemic hyperinsulinaemic clamp method [22, 23] showed decreased glucose infusion rate in acromegalic patients. In addition, Kasayama et al. revealed that the insulin sensitivity was reduced in acromegalic patients with normal glucose tolerance [15].

Previous studies considered that the acromegalic patients with NGT had the same beta-cell function with healthy subjects. However, our study showed a compensatory upregulation of insulin secretion in newly diagnosed acromegaly with NGT.  Figure S1 shows that the beta-cell sensitivity-secretion hyperbolas of acromegalic patients with NGT are lower than those of normal people, whether under fasting or after glucose load. In another word, the beta-cell function (DI) in acromegaly with NGT was impaired. HOMA-beta may be exaggerated to evaluate *β*-cell function since insulin secretion is stimulated by higher glucose in acromegaly. However, the disposition index which represents insulin sensitivity-corrected *β*-cell function (e.g., ISSI2) reflects *β*-cell function reserve and is more objective. It is a quantitative, convenient, and accurate tool in analyzing epidemiologic data and identifying incipient *β*-cell dysfunction [24]. As the OGTT and insulin releasing test showed, the beta-cell compensatory secretion is not sufficient to inhibit blood glucose elevation, and blood glucose in the acromegaly group is higher than the normal group and peaks at 60 min and lasts for 120 min.

Studies have revealed the possible mechanism of decreased insulin sensitivity. Karlander et al. showed that hepatic glucose production was higher than normal in acromegalic subjects under fasting condition despite hyperinsulinemia [25]. Galbraith et al. demonstrated reduced glucose uptake in the forearm tissues after intra-arterial insulin administration in acromegaly [26]. Foss et al. showed that forearm muscle glucose uptake and nonoxidative metabolism after the ingestion of 75 g glucose were impaired in acromegaly [27]. Balbis et al. supposed alterations in the insulin receptor number and in the tyrosine kinase activity develop in response to changes in insulin levels [28].

The current study combined CGMS technology with newly diagnosed nonintervention acromegaly patients to observe the 24-hour glucose profile of patients with high GH levels. According to the dynamic blood glucose monitoring, average blood glucose of patients with acromegaly was significantly higher than that of the control group (Figure 3). There is greater glycemic variability in those with acromegaly compared with healthy controls according to Figure 3, but it did not reach statistical significance. There may be some reasons to explain this paradox. One possible reason is that overall blood glucose of acromegaly patients is higher than that of healthy people, but the glucose variability of patients with acromegaly has not changed significantly yet. Another reason is the small sample size.

The data in this study show that the traditional metrics to diagnose diabetes and prediabetes, i.e., fasting glucose, HbA1c, and OGTT, do not capture the significant dysglycemia that patients with acromegaly have. This finding is supported by other studies that have used CGM to uncover similar dichotomies between traditional approaches and CGM approaches [29, 30]. Salkind et al. saw this in patients with morbid obesity who would otherwise be characterized as completely normal [29]. A similar finding has been published in patients with cystic fibrosis [30]. Our study has verified that CGM is a sensitive technology that may surpass the traditional ones in identifying dysglycemia and then, perhaps, lead to appropriate interventions.

In conclusion, our research suggests that newly diagnosed acromegalic patients with NGT were characterized by insulin resistance and impaired *β*-cell function after glucose load. CGM shows that MBG of NGT acromegaly patients is higher than that of normal people.

## Figures and Tables

**Table 1:** General characteristics of the study participants.

	Acromegaly	Control	*P*
*n*	8	8	
Gender (male/female)	2/6	2/6	
Age (years)	39.3 ± 9.6	38.0 ± 9.6	0.799
BMI (kg/m^2^)	24.69 ± 2.03	23.43 ± 2.73	0.311
HbA1c (%)	5.73 ± 0.30	5.35 ± 0.23	0.025
Nadir GH on OGTT (*μ*g/L)	6.39 (2.73–12.12)	0.34 (0.12–0.48)	0.001
IGF-1 index	2.35 ± 0.42	0.62 ± 0.19	<0.001
BG_0_ (mmol/L)	5.14 ± 0.19	4.91 ± 0.38	0.156
BG_120_ (mmol/L)	6.63 ± 0.50	4.98 ± 1.43	0.014
INS_0_ (mU/L)	12.85 (12.33–23.43)	10.85 (6.28–13.00)	0.189
INS_120_ (mU/L)	112.85 (46.78–175.45)	34.05 (17.10–55.55)	0.009

Data are means ± SD or medians (25^th^–75^th^ percentile). BMI: body mass index; BG_0_ and BG_120_: blood glucose levels in fasting and 120 min during OGTT. INS_0_ and INS_120_: insulin levels in fasting and 120 min during OGTT; IGF-1 index = IGF-1/age-appropriate upper limit of normal range (ULN).

**Table 2:** The insulin sensitivity and beta-cell function of newly diagnosed acromegalic patients.

	Acromegaly	Control	*P*
HOMA-beta	159.77 (139.51–294.86)	117.96 (98.43–220.97)	0.172
HOMA-IR	2.95 (2.76–5.35)	2.36 (1.31–3.15)	0.059
BG_0_/INS_0_	0.39 (0.23–0.43)	0.52 (0.36–0.76)	0.103
HOMA-beta × (BG_0_/INS_0_)	61.18 (58.89–68.44)	74.06 (61.17–82.65)	0.114
(INS_30_ − INS_0_)/(BG_30_ − BG_0_)	26.51 (16.54–71.40)	22.50 (14.90–42.57)	0.674
AUC_INS_/AUC_BG_	11.23 (8.90–24.02)	7.07 (5.56–10.99)	0.074
IS_OGTT_	50.25 (34.76–69.50)	104.63 (67.14–129.58)	0.012
ISSI2	551.32 (520.87–684.55)	716.12 (588.83–779.83)	0.046

Data are medians (25^th^–75^th^ percentile). AUC_BG_: area under the glucose curve; AUC_INS_: area under the insulin curve; AUC_INS_/AUC_BG_: insulin secretion index; IS_OGTT_: insulin sensitivity after glucose load; ISSI2: insulin secretion-sensitivity index-2.

**Table 3:** The glucose profile and glycemic variability of newly diagnosed acromegalic patients.

	Acromegaly	Control	*P*
MBG (mmol/L)	6.46 ± 0.44	5.31 ± 0.49	<0.001
PT7.8 (%)	8.13 ± 6.49	1.75 ± 2.66	0.022
PT3.9 (%)	2.38 ± 4.60	6.13 ± 7.43	0.249
SDBG (mmol/L)	0.85 ± 0.40	0.60 ± 0.18	0.127
MAGE (mmol/L)	2.44 ± 1.61	1.59 ± 0.58	0.180
CV (%)	12.96 ± 5.27	11.18 ± 3.11	0.424

MBG: mean blood glucose; PT7.8: percentage of time (PT) for blood glucose ≥ 7.8 mmol/L; PT3.9: percentage of time (PT) for blood glucose ≤ 3.9 mmol/L; SDBG: standard deviation of blood glucose; MAGE: mean amplitude of glycemic excursions; CV = SDBG/MBG.

## Data Availability

The data used to support the findings of this study are available from the corresponding author upon request.

## Supplementary Materials

Supplementary Materials Figure S1: beta-cell sensitivity-secretion hyperbola under fasting and after glucose load. A: under fasting; B: after glucose load.
