# Supplementary figures and images for: The Beta-Cell Function and Glucose Profile of Newly Diagnosed Acromegalic Patients with Normal Glucose Tolerance
Source: Int J Endocrinol. 2021 Dec 7;2021:3666692. doi: 10.1155/2021/3666692 (PMC8670947; doi:10.1155/2021/3666692)

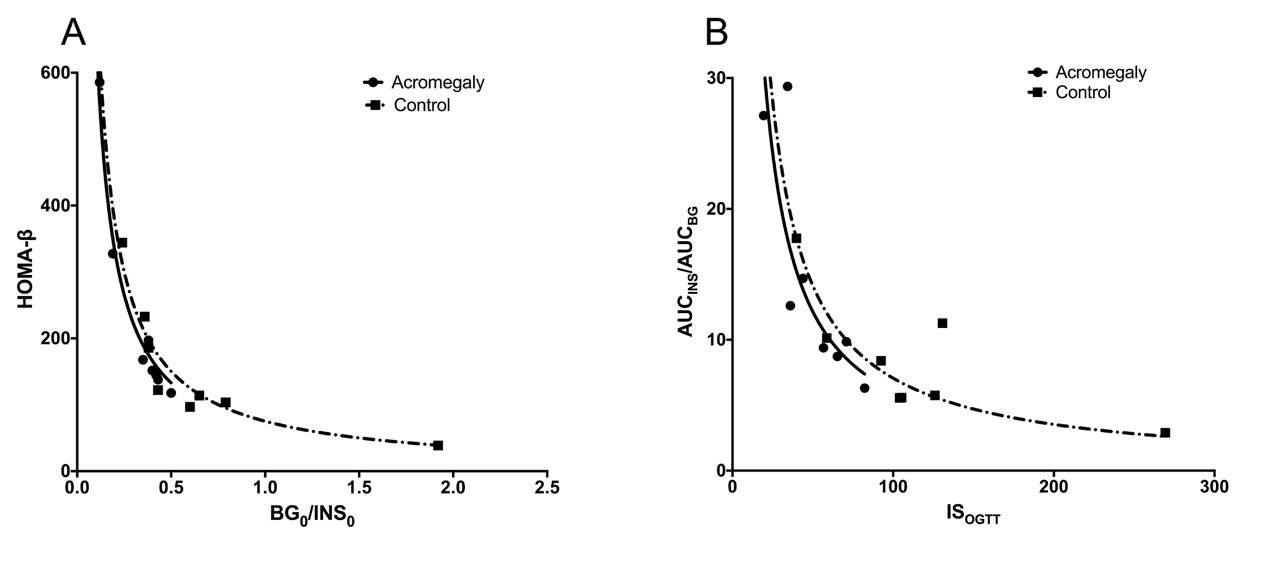

Supplement: Supplementary Materials — Figure S1: beta-cell sensitivity-secretion hyperbola under fasting and after glucose load. A: under fasting; B: after glucose load. [file 3666692.f1.zip › 3666692.f1/Figure S1 (1).jpg]
